# The Role of Cholesterol and Fatty Acids in the Etiology and Diagnosis of Autism Spectrum Disorders

**DOI:** 10.3390/ijms22073550

**Published:** 2021-03-29

**Authors:** Cecilia Maria Esposito, Massimiliano Buoli, Valentina Ciappolino, Carlo Agostoni, Paolo Brambilla

**Affiliations:** 1 Department of Neurosciences and Mental Health, Fondazione IRCCS Ca’Granda Ospedale Maggiore Policlinico, Via F. Sforza 35, 20122 Milan, Italy; cecilia.esposito@unimi.it (C.M.E.); massimiliano.buoli@unimi.it (M.B.); valentina.ciappolino@policlinico.mi.it (V.C.); paolo.brambilla1@unimi.it (P.B.); 2 Department of Pathophysiology and Transplantation, University of Milan, 20122 Milan, Italy; 3 Department of Clinical Sciences and Community Health, University of Milan, 20122 Milan, Italy; 4 Pediatric Unit, Fondazione IRCCS Ca’ Granda Ospedale Maggiore Policlinico, 20122 Milan, Italy

**Keywords:** autism spectrum disorders, cholesterol, fatty acids, Asperger syndrome, pathogenesis

## Abstract

Autism spectrum disorders (ASDs) are a group of neurodevelopmental disorders whose pathogenesis seems to be related to an imbalance of excitatory and inhibitory synapses, which leads to disrupted connectivity during brain development. Among the various biomarkers that have been evaluated in the last years, metabolic factors represent a bridge between genetic vulnerability and environmental aspects. In particular, cholesterol homeostasis and circulating fatty acids seem to be involved in the pathogenesis of ASDs, both through the contribute in the stabilization of cell membranes and the modulation of inflammatory factors. The purpose of the present review is to summarize the available data about the role of cholesterol and fatty acids, mainly long-chain ones, in the onset of ASDs. A bibliographic research on the main databases was performed and 36 studies were included in our review. Most of the studies document a correlation between ASDs and hypocholesterolemia, while the results concerning circulating fatty acids are less univocal. Even though further studies are necessary to confirm the available data, the metabolic biomarkers open to new treatment options such as the modulation of the lipid pattern through the diet.

## 1. Introduction

Autism spectrum disorders (ASDs) are a group of neurodevelopmental disorders which have their onset in childhood and include autistic disorder, Asperger syndrome, childhood disintegrative disorder and pervasive developmental disorder not otherwise specified [1]. Nevertheless, the classification of ASDs has undergone changes over the years with significant changes between DSM-5 and the previous versions of the manual. The whole spectrum affects the general population with a prevalence of 1–5% and with a predominance of male gender (Lord et al., 2018). The core symptoms include the alteration of social cognition, deficits in social interaction and impaired communication abilities [2,3,4]. Children with ASDs also show atypical behaviors, which tend to be repetitive, ranging from stereotypies to perseverations, both in general and in relation to eating behaviors [5,6,7]. The restricted pattern of behavior also applies to the field of interests, which tends to be characterized by narrowness and repetitiveness [8]. Moreover, in 30% of cases, ASDs co-occur with intellectual disability, a factor that worsens prognosis [9].

Given the variability of the clinical manifestations and the diagnostic delay that often occurs in patients affected by ASDs, the detection of biological biomarkers capable of facilitating early diagnosis appears of extreme importance [6,10,11,12]. Screening campaigns have been applied to facilitate the diagnosis of ASDs; however, they did not result to be successful being essentially based on clinical phenotypes that have the limit of variable specificity and prognostic value [13,14]. The identification of reliable biomarkers could improve the limits of clinical diagnosis and it is not a coincidence that most research on this field has focused on biological aspects in the last years. The candidate markers regard different biological systems including genetics, immunity, brain changes and metabolism [15,16,17]. Among these, the metabolic hypothesis is particularly promising as it integrates genetic vulnerability with environmental aspects [18,19,20].

ASDs seem to be the result of an imbalance between excitatory and inhibitory synapses and of a dysfunction in the synaptic plasticity of the brain, which leads to a disrupted connectivity during the brain development [21,22,23,24,25]. Cholesterol homeostasis was demonstrated to be involved in correct synaptic functioning and consequently this molecule was hypothesized to play a role in the etiology of ASDs [26,27]. Of note, several evidences support the involvement of an aberrant metabolism of cholesterol and fatty acids in the pathogenesis of autism. Firstly, polymorphisms of genes, codifying for molecules involved in mevalonate/cholesterol metabolism, were associated with an increased risk of ASDs [28]. In addition, subjects suffering from Smith-Lemli-Opitz Syndrome (SLOS), a genetic disorder implying a deficit in cholesterol biosynthesis, appear to be predisposed to develop ASDs [26]. Finally, the regulation of synaptic excitation seems to be connected to the allosteric modulation of N-Methyl-D-Aspartate Receptors (NMDARs), mediated by 24(S)-hydroxycholesterol (OHC) that is the most relevant brain cholesterol metabolite [29].

Cholesterol is transported through the blood by lipoproteins, where it is esterified with fatty acids [30]. Lipoproteins contain also phospholipids carrying further fatty acids. Circulating fatty acids are in continuous exchange with membrane lipids, in order to reach a biologic balance, specific for any individual and based on genetic and environmental factors (mainly dietary habits) [31]. Long-chain polyunsaturated fatty acids (namely, arachidonic acid [AA] and docosahexaenoic acid [DHA]) are the biologically most active compounds within the fatty acid family. AA and DHA might influence the neural response and their concentrations in the brain may largely vary, thus influencing behavioral domains [32]. Eicosapentaenoic acid (EPA), the biochemical precursor of DHA, even if not directly present in neural membranes, contributes to the AA/DHA balance and it plays a role in modulating inflammation [18]. 

If the metabolism of cholesterol and fatty acids plays a role in the onset and severity of ASDs, the modulation of diet appears to be promising for the prevention or management of these disorders. Steps have been already moved towards this direction and several studies hypothesized the role of specific dietary interventions, such as limiting and/or depriving either gluten and casein supply, even in association with ketogenic diets [33,34]. Changes in type of diet, by modifying the intestinal microbiome, could have anti-inflammatory properties which therefore would be beneficial for the Central Nervous System [35,36,37]. Even though the data regarding these therapeutic options are preliminary, they appear promising especially as they regard a non-pharmacological approach for improving symptoms. Furthermore, the contribution of diet in the onset of ASD has been poorly investigated till now, especially as regards the lipid component, but it is a promising field of investigation. As a matter of fact, few authors evaluated the impact of diet modifications on the course of ASDs, but the results appear to be promising despite being preliminary [6,18]. Starting from these assumptions, the purpose of the present review is to summarize the available data about the role of cholesterol and fatty acids, mainly long-chain ones, in the onset of ASDs, respectively.

## 2. Methods

A search in the main databases (PubMed, Embase, PsycInfo, Isi Web of Knowledge, Medscape, The Cochrane Library) was conducted with last check on 31 October 2020. The terms “autism”, “Asperger” were individually matched with “cholesterol”, “fatty acids”, “arachidonic acid”, “EPA” and “DHA”.

**Figure 1:**
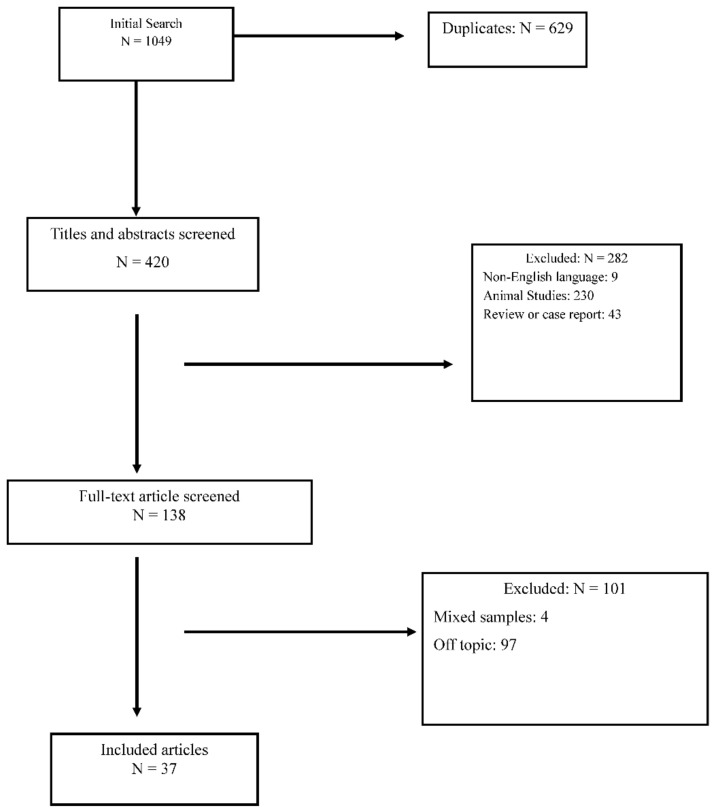
Prisma Diagram for reviews.

The initial search was obtained using only headings. Then, a double-blind selection of papers (MB and CME) was manually performed, to select the articles suiting with the topic of this review. The selection process is detailed in Figure 1. Studies concerning the role of cholesterol and fatty acids in the pathogenesis of ASDs were included. Therefore, studies focusing on therapeutic strategies were excluded, as well as those in which the sample of patients with ASD was not analyzed individually but only in a context of a mixed sample. Furthermore, exclusion criteria consisted of: (1) animal studies; (2) case reports and reviews; (3) use of fatty acids as parallel treatments; (4) maternal diet studies; (5) intestinal microbiome studies. Moreover, we excluded paper in a language different from English. 

One thousand and forty nine papers were initially identified, of which 1012 were discarded because they were duplicates or because they met exclusion criteria. Thirty-seven papers satisfied the inclusion criteria. Table 1 summarizes the results of the included papers.

## 3. Results

### 3.1. ASDs and Cholesterol

As regards the relationship between ASDs and cholesterol, 14 studies were identified, conducted between 2001 and 2020. Among the identified studies, most of them regard the association between ASDs and hypo- or hypercholesterolemia and they report discordant results, as highlighted in Table 2.

Several studies reported the association between ASDs and hypocholesterolemia [40,45,70,71]. A recent study by Benhachenhou and colleagues showed that in subjects affected by ASDs the prevalence of hypocholesterolemia was more than threefold higher than in the general population [70]. Total cholesterol (TC) levels below the 10th percentile would be also associated with a higher risk of comorbidities such as intellectual disability and the presence of anxious-depressive symptoms [70]. In addition to TC levels, low-density lipoprotein (LDL) cholesterol levels were also found to be reduced in patients with autism compared to the general population [42,67]. Of note, Dziobeck and collaborators detected reduced levels of LDL cholesterol in face of increased levels of TC in patients with Asperger syndrome [42]. Consequently, an interesting hypothesis is that impaired cholesterol synthesis could affect its concentration in the central nervous system in subjects affected by ASDs. In support to this speculation, red blood cell (RBC) membranes of children affected by autism have on the average significantly less cholesterol and significantly more ganglioside 1 (GM1), which is involved in neuronal plasticity, than RBC membranes of healthy controls [38]. On the other hand, the association between SLOS, a genetic disorder caused by a defect in cholesterol biosynthesis, and autism demonstrates the importance of the entire lipid panel in the etiology of ASDs [52]. The clinical picture of SLOS consists of hypocholesterolemia and abnormal accumulation of the precursor 7-dehydrocholesterol (7-DHC) [40], however, neither TC plasma levels, nor 7-DHC levels, nor 8-dehydrocholesterol (8-DHC) levels appear to correlate with the presence of autistic symptoms in SLOS patients [43]. 

In contrast with the mentioned results, a study conducted by Blazewicz and collaborators showed an increase of TC levels in adolescents with autism, divided into groups according to dietary characteristics [33]. In particular, the presence of increased TC/high-density lipoprotein (HDL) plasma levels was found in patients with ASDs compared to healthy controls regardless of the type of diet. In addition, the groups of patients characterized by low-fat diet or regular diet showed an increase in triglycerides (TG), while the group of patients following a gluten-casein-free diet exhibited an increase in non-HDL cholesterol [33].

Other studies, on the contrary, did not identify statistically significant differences in patients with ASDs compared to healthy controls, neither with regard to TC and LDL cholesterol levels [49,56], nor with regard to the levels of cholesterol sulfate (CS), which is an endogenous steroid involved in the stabilization of plasma membranes [53]. Kim and colleagues, however, identified higher LDL/HDL ratio in ASDs patients than controls, as well as the association between autism and plasma TG, HDL and LDL/HDL ratio [49].

### 3.2. ASDs and Fatty Acids

It was possible to identify 17 articles concerning the association between ASDs and fatty acids in general, 5 articles concerning the association between ASDs and AA, 7 articles concerning the association between ASDs and EPA, 11 articles concerning the association between ASDs and DHA. Among these manuscripts, 23 resulted to be duplicates.

Bell and colleagues reported a higher frequency of fatty acids deficiency (FAD) in patients with autism and Asperger syndrome compared to healthy controls [41]. Specifically, patients with regressive autism, which includes individuals who passed all developmental criteria up to 18–36 months and thereafter regressed into autism, had higher percentages of stearic acid (18:0), linoleic acid (18:2*n* − 6) and total saturates in their RBC membranes than controls, as well as higher lignoceric acid (24:0), docosapentaenoic acid (22:5*n* − 6), nervonic acid (24:1) and AA/EPA ratio; these latter characteristics are shared by regressive autism and Asperger syndrome [41]. Furthermore, patients with regressive autism showed lower oleic acid (18:1*n* − 9) and AA values and patients both with regressive autism and with Asperger syndrome presented lower docosapentaenoic acid and total *n* − 3 polyunsaturated fatty acids (*n* − 3 PUFAs) compared to controls [41]. A study then analyzed the erythrocyte and plasma fatty acid compositions of children with autism compared to healthy controls and children with developmental delay, showing decreased levels of lignoceric acid (24:0) and nervonic acid (24:1) in children with developmental delay with respect to ASDs [47]. According to Bell and colleagues, patients with ASDs would not have an underlying specific “phospholipid disorder”, but, within the phospholipid pool, an increase of the AA/EPA ratio would suggest the presence of an imbalance of essential highly unsaturated fatty acids in some autistic children [47]. According to Howsmon and colleagues, on the other hand, the erythrocyte fatty acid profiles are not reliable markers that can be applied for the classification of subjects affected by ASDs [68].

On the contrary, Brown and colleagues identified essential fatty acids (EFAs) deficiencies in patients with ASDs, giving rise to the hypothesis that a dysregulation of the fatty acid phospholipid metabolism plays an essential role in the etiology of these conditions [55]. Furthermore, other studies reported abnormal concentrations of linoleic and α-linolenic acids, higher levels of DHA, AA and lower levels of phospholipids in ASDs compared to controls [44,51,58,61,62,73], as well as the increase in most of the saturated fatty acids and a decrease in most of polyunsaturated fatty acids [50]. As reported in Table 3, an elevated Omega-6 (ω6)/Omega-3 (ω3) ratio was found by different authors [39,54,58], where ω3 and ω6 are a group of essential fatty acids involved in maintaining the integrity of plasma membranes. DHA and EPA belong to the ω3 group, while linoleic acid and AA belong to the ω6 group. The finding of increased monounsaturated fatty acids, decreased EPA and DHA and consequently of an increased ω6/ω3 ratio could explain the reduction of erythrocyte membrane fluidity in ASDs compared to controls [54]. Reduced levels of DHA, EPA and AA in the RBCs as well as reduced levels of DHA, AA, EPA, α-linolenic and linoleic acids in plasma were identified in ASDs patients than healthy controls [44,46,48,57,59,60,63,65,66]. Furthermore, the DHA/AA ratio was supposed to be a reliable marker to differentiate subjects affected by ASDs from healthy individuals [61]. The results about the association between ASDs and DHA, AA and EPA serum/plasma levels are summarized in Table 3, Table 4, Table 5 and Table 6, respectively. Finally, lower serum levels of very-low-density lipoproteins (VLDL) and apoprotein B (APOB) were detected in serum of children with ASDs than typical developmental controls [72]. 

According to Puig-Alcatraz and colleagues, no significant differences were observed in adipic acid and suberic acid levels in patients with ASDs compared to healthy controls [64]. However, the increase of adipic acid levels was indirectly correlated with the severity of the deficit in socialization and communication skills in children with an ASD [64]. Plasma levels of fatty acids (both ω3 and ω6 ones) were found to be positively correlated with social interaction of children with ASDs [72]. The findings by Bell and colleagues would allow to differentiate the clinical subgroups of patients affected by ASDs: for example, patients with regressive autism showed a reduction in highly unsaturated fatty acid levels in polar lipids, after storage of RBC at −20 °C for 6 weeks, while this reduction was not observable neither in controls nor in patients with classical autism or Asperger syndrome [41]. Patients presenting with both autism and Asperger syndrome exhibited increased EPA and DHA levels and decreased AA, 20:3*n* − 6 levels and AA/EPA ratio in their RBC polar lipids, after EPA-rich fish oil supplementation. These patients presented also higher concentrations of type IV phospholipase A2 in RBCs compared to controls [41].

## 4. Discussion

Several studies indicate that abnormalities in the lipid panel are usual in patients with ASDs and that the degree of these alterations may affect the severity of clinical symptoms [64,72]. While we are probably still far from having reliable lipid biomarkers to be used for early diagnosis of these disorders often characterized by an insidious onset, available findings seem to be promising towards this direction.

Specifically, most observational studies detected an association between low cholesterol plasma levels and ASDs [40,45,70,71]. This evidence is supported by the cases of SLOS, in which the deficit of cholesterol biosynthesis is associated with clinical manifestations largely overlapping with a diagnosis of autism [26,38]. The most accredited hypothesis claims that there is a correspondence between low plasma cholesterol levels and its reduction in the central nervous system, with a consequent alteration in the lipid constitution of neuron membranes [52]. This abnormality would lead to an imbalance of excitatory and inhibitory synapses and to a dysfunction in the synaptic plasticity of the brain [22,24,74]. Another interesting hypothesis is that the alteration of cholesterol and its metabolites, such as OHC, can be involved in the pathogenesis of ASDs by inducing oxidative stress and consequent glutamate toxicity at neuronal level [75]. The consequence would be a dysfunctional brain connectivity, an altered neurodevelopment and the onset of ASDs [24]. It should be also emphasized that the role of cholesterol in psychiatric disorders has not been fully understood and that the association with its plasma levels varies according to the disorder or psychiatric symptoms. While low cholesterol plasma levels were found to be associated with suicide attempts, particularly violent ones (similarly to ASDs) [76], depressive symptoms seem to be associated with an increase of plasma cholesterol levels [77]. This is not surprising as autism and depression are at the opposite symptomatic poles (e.g., active social avoidance in ASDs versus passive social withdrawal in depression) [78]. In addition, psychiatric symptoms can modulate the function of second messengers or enzymes involved in lipid metabolism. Of note, a recent article demonstrated that depressive symptoms were directly associated with levels of Proprotein Convertase Subtilisin/Kexin Type 9 (PCSK9), an enzyme that regulates the degradation of LDL receptor [79].

With regard to fatty acids, the results are more discordant within highly heterogeneous settings of observations. In general, it would seem that patients with ASDs show a trend towards lower levels of both DHA and AA in comparison with healthy controls [46,48,54,57,59,60,61,63,65], as well as an elevated ω6/ω3 ratio [39,54,58,60]. Again, these alterations support the hypothesis of an inflammatory dysregulation in patients affected by psychiatric conditions [80,81,82] including ASDs [83]. In addition, it was demonstrated that changes in lipid profile may enhance systemic and brain oxidative stress affecting synaptic function [75,84], paving the way for new therapeutic perspectives [18].

Taken as a whole, increasing evidence seems to support a role of cholesterol and probably fatty acids in the etiology of ASDs. These findings open up the possibility of an environmental modulation of lipid alterations in ASDs, for example through the diet. In this sense, various dietary supplementation treatments in patients with ASDs were tested with promising results [34,35]. Of note, the very few clinical trials included in the present review regard attempts to modify the lipid panel, either through diet or through dietary supplements [33,41,46]. Meguild and colleagues remarked the role of fish oil supplementation in increasing plasma levels of DHA, as well as in improving the clinical symptoms of subjects affected by autism [46]. On the other hand, Blazewicz and collaborators identified a specific correlation between nutritional habits and plasma cholesterol levels (TC, HDL and non-HDL cholesterol) [33]. These studies open the perspective of modifying diet styles to improve the prognosis of patients with ASDs. 

Several clinical trials currently underway are based precisely on the hypothesis of the metabolic etiology of the ASDs and analyze in particular the possibilities of intervention on the nutritional side. In particular, trials were carried out to test specific diets, such as the ketogenic diet or the gluten-free one, as potential modifiers of the clinical course of patients affected by ASDs [33,34]. The pathogenetic hypothesis underlying these attempts is essentially based on the possibility of modulating the intestinal microbiome, which in recent years has been recognized as a factor implicated in the onset of psychiatric symptoms [36]. In fact, through the diet or through the use of probiotics it is possible to modify the intestinal microbiome and, thus, its critical role in the regulation of immunity and brain function [35,37].

Major limitations of the included studies are:

(1) a high heterogeneity of study designs, with a wide range of sample sizes, involving in some cases very small groups, so largely limiting the statistical power (rarely reported by authors);
(2) differences in the type of cell or biological material (e.g., RBCs or plasma) used to measure the lipid/fatty acid profile;
(3) the possible influence of concurrent pharmacological treatment on lipid levels and other potential confounding factors, including the concomitance of psychiatric or medical comorbidity;
(4) the heterogeneity in the number of fatty acids tested and/or described in the different reports.

Finally, the high number of observational reports does not allow the exclusion of a possible reverse causality until more controlled clinical trials are available. For all these reasons, the data reported in this article have to be considered as preliminary and need to be confirmed by further research studies.

A final aspect to underline is the predominance of male sex in the samples of the included studies, in agreement with the available literature [85]. The present manuscript had not as purpose the evaluation of the impact of gender on lipid profile of subjects affected by ASDs; however, this aspect could be investigated by future meta-regression analyses. In conclusion, to better understand the role of cholesterol and fatty acids in the pathogenesis of ASDs, it would be helpful to enrich the available literature with studies having controlled designs, with reliable sample sizes and long-term follow-up.

## Figures and Tables

**Table 1:** Summary of the results of the selected studies about the role of cholesterol and fatty acids in the pathogenesis of ASDs.

Study	Design	Sample	Intervention	Biomarkers	Findings in ASDs
Tierney et al., 2001 [38]	Cross-sectional study	28 SLOS children	/	Plasma TC	SLOS, which presents with hypocholesterolemia, is associated with the presence of autism.
Vancassel et al., 2001 [39]	Cross-sectional study	15 ASD subjects: Mean age 8.4 years 73.33% males 18 ID subjects: Mean age 8.8 years 72.22% males	/	Plasma FA	A marked reduction in the levels of DHA (23%) was shown in ASD, resulting in significantly lower levels of *n* − 3 PUFA (20%; *p* = 0.032) and a significant increase in the ω6/ω3 ratio (25%; *p* = 0.039).
Goldenberg et al., 2002 [40]	Cross-sectional study	45 SLOS subjects	/	Plasma TC, 7-DHC, 8-DHC	Plasma hypocholesterolemia correlates significantly with clinical severity (1–2.5% increased risk).
Bell et al., 2004 [41]	Cross-sectional study	ASD children HC	/	Plasma and RBC membranes FA composition	Higher frequency of FAD was found in patients with autism and Asperger syndrome compared to HC. Patients with regressive autism had higher percentages of stearic acid, linoleic acid and total saturates in their RBC membranes than HC, as well as higher lignoceric acid, docosapentaenoic acid, nervonic acid and AA/EPA ratio; these latter characteristics are shared by regressive autism and Asperger syndrome. Patients with regressive autism showed lower oleic acid and AA values and patients both with regressive autism and with Asperger syndrome presented lower docosapentaenoic acid and total *n* − 3 PUFAs compared to HC.
Dziobek et al., 2006 [42]	Cross-sectional study	22 Asperger children: Mean age 40.8 ± 10.8 years 77.3% male 22 HC: Mean age 44.6 ± 14.8 years 77.3% male	/	Plasma TC, LDL-C, TG	Asperger subjects showed statistically significant elevated levels of TC, LDL-C and TG and significant lower levels of HDL compared with HC. After controlling for physical activity, group differences remained significant for TC (*p* = 0.016) and LDL-C (*p* = 0.017), but not for HDL-C (*p* = 0.323) and TG (*p* = 0.217).
Sikora et al., 2006 [43]	Clinical trial	14 SLOS children: Mean age 7.1 ± 3.5 years	2-years cholesterol supplementation	Plasma TC, 7-DHC, 8-DHC	Plasma TC, 7-DHC and 8-DHC at baseline and after supplementation did not correlate with the presence or severity of autistic symptoms.
Sliwinski et al., 2006 [44]	Cross-sectional study	16 high-functioning ASD subjects aged 12–18 years: 100% males 22 HC	/	Plasma FA	In ASD there was a significant increase in the fraction of DHA and an increase in the total ω3/ω6 ratio.
Tierney et al., 2006 [45]	Cross-sectional study	100 ASD children	/	Plasma TC, 7-DHC	Of 19% of subjects with low TC (<100 mg/dL), 31.5% met criteria for ASD.
Meguild et al., 2008 [46]	Clinical trial	30 ASD children aged 3–11 years: 60% males 30 age- and sex-matched HC	3-months fish oil supplementation	Plasma FA	First assessment: In ASD group, linolenic acid showed a significant reduction (71%), followed by DHA (65%) and AA (45%), while linoleic acid was the least affected PUFA (32%), compared to HC. Second assessment: 66% of autistic children showed clinical and biochemical improvement (decrease in Childhood Autism Rating Scale scores: *p* < 0.0001). Plasma PUFA showed increase in plasma levels after supplementation (all *p* < 0.0001).
Bell et al., 2010 [47]	Cross-sectional study	49 ASD children: Mean age 7.5 ± 3.5 years 89.80% males 39 DD children: Mean age 6.0 ± 3.3 years 89.74% males 52 HC: Mean age 7.5 ± 3.6 years 94.23% males	/	Plasma and RBC membranes FA composition	RBC and plasma FA in ASD had an increased AA/EPA ratio. Decreased levels of lignoceric acid and nervonic acid were found in children with DD with respect to ASD. ASD subjects consuming fish oil showed reduced erythrocyte AA, adrenic acid, docosapentanaeoic acid and total *n* − 6 FA and increased EPA, DHA and total *n* − 3 FA along with reduced *n* − 6/*n* − 3 and AA/EPA ratios.
Wiest et al., 2009 [48]	Cross-sectional study	153 ASD children: Mean age 3.75 years 88.89% males 97 HC: Mean age 3.42 years 78.36% males	/	Plasma lipidomics	Levels of CE, FS, PC and FA did not differ between ASD and HC. Total TG, lysophosphatidylcholine, PE and diglyceride levels were higher in the ASD than the HC group (respectively, *p* = 0.006; *p* = 0.001; *p* = 0.007; *p* = 0.005). DHA was significantly decreased in ASD with respect to HC (*p* < 0.05).
Kim et al., 2010 [49]	Cross-sectional study	29 ASD children: Mean age, 10.1 ± 1.3 years 100% male 29 age- and sex-matched HC	/	Plasma TC, HDL-C, LDL-C, LDL/HDL ratio, TG	The mean TG level was significantly higher, whereas the mean HDL-C level was significantly lower in cases as compared to controls (respectively, *p* = 0.01; *p* = 0.003). There was no significant difference in TC and LDL-C levels between ASD and HC. The LDL/HDL ratio was significantly higher in ASD compared to HC (*p* = 0.03). Autism was associated with plasma TG, HDL and the LDL/HDL ratio (respectively, *p* = 0.01; *p* = 0.0003; *p* = 0.04).
El-Ansary et al., 2011 [50]	Cross-sectional study	25 ASD children aged 4–12 years 16 HC aged 4–11 years	/	Plasma FA, PE, PS and PC	ASD patients showed higher LA/AA, ALA/DHA, AA/DHA, EPA/AA ratios compared to HC (respectively, *p* = 0.034; *p* = 0.004; *p* = 0.000; *p* = 0.000). ASD patients showed higher plasma PE, PS and PC compared to HC (respectively, *p* = 0.002; *p* = 0.000; *p* = 0.000).
El-Ansary et al., 2011 [51]	Cross-sectional study	26 ASD children aged 4–12 years 26 age- and sex-matched HC	/	Plasma FA	ASD patients showed an increase in acetic, valeric, hexanoic and stearidonic acid compared to HC (respectively *p* = 0.000; *p* = 0.000; *p* = 0.000; *p* = 0.009). ASD subjects show different percentage decrease of saturated acids (propionic, butyric, caprylic, decanoic, lauric, palmitic and stearic) together with mono (oleic) and polyunsaturated fatty acids (arachidic, α-linolenic, DHA, linoleic, γ-linolenic, AA, elaidic) compared to HC (respectively, *p* = 0.000; *p* = 0.028; *p* = 0.000; *p* = 0.000; *p* = 0.000; *p* = 0.0037; *p* = 0.000; *p* = 0.000; *p* = 0.000; *p* = 0.0045; *p* = 0.000; *p* = 0.023; *p* = 0.000; *p* = 0.000; *p* = 0.000).
Schengrund et al., 2012 [52]	Cross-sectional study	16 ASD children: Mean age 5.13 years 93.75% male 20 HC: Mean age 6.0 years 40% male	/	RBC membranes composition: GM1 and cholesterol	ASDs children have less cholesterol and more GM1 in their RBC membranes than HC (respectively, *p* = 0.012; *p* = 0.019).
Fong et al., 2013 [53]	Cross-sectional study	102 ASD children: Mean age 3.5 years 52.0% male 102 HC: Mean age 3.9 years 52.0% male	/	Plasma CS	Comparison of normal and autistic children showed no statistically significant difference in plasma CS level.
Ghezzo et al., 2013 [54]	Cross-sectional study	25 ASD children: Mean age 7.8 ± 2.23 years 80.95% males 23 HC: Mean age 7.6 ±1.96 years 70% males	/	RBC membranes FA composition	Alteration in RBC FA membrane profile (increase in monounsaturated fatty acids, decrease in EPA and DHA with a consequent increase in ω6/ω3 ratio) were found in ASD compared to HC (respectively, *p* < 0.01; *p* < 0.05; *p* < 0.01).
Brown et al., 2014 [55]	Cross-sectional study	19 ASD children 23 HC (siblings)	/	Plasma FA	Those infants not breastfed (with colostrum) within the first hour of life and who had a history of FAD symptoms were more likely to have an ASD diagnosis.
Moses et al., 2014 [56]	Cross-sectional study	80 adults with ASD 77 adults with ID 828 HC	/	Plasma TC	TC levels of people with ASD and ID were significantly lower than those of HC (*p* < 0.001) but after adjusting for gender, age and BMI and using Bonferroni correction, the significance was lost.
Brigandi et al., 2015 [57]	Cross-sectional study	121 ASD subjects aged 3–17 years 110 HC aged 3–17 years	/	Plasma and RBC membranes FA composition	The percentage of total PUFA was lower in ASD than in HC; levels of AA and DHA were particularly decreased (*p* < 0.001).
Esparham et al., 2015 [58]	Cross-sectional study	7 ASD children aged 7–18 years: 71.43% males	/	Plasma FA	An abnormal level of α-linolenic, linoleic acid and high levels of DHA were found, as well as an elevated ω6/ω3 ratio.
Jory, 2015 [59]	Cross-sectional study	11 ASD children: Mean age 3.05 ± 0.79 years 72.73% males 15 HC: Mean age 3.87 ± 1.06 years 40% males	/	Plasma and RBC membranes FA composition	Children with ASD demonstrated lower RBC DHA, EPA, AA and ω3/ω6 ratios (respectively, *p* < 0.0003; *p* < 0.03; *p* < 0.002; *p* < 0.001). They also demonstrated lower plasma DHA, AA and linoleic acid levels (respectively, *p* < 0.02; *p* < 0.05; *p* < 0.02).
Mostafa and Al-Ayadi, 2015 [60]	Cross-sectional study	100 ASD children: Mean age 6.22 ± 2.1 years 78% males 100 HC: Mean age 5.96 ± 2 years 78% males	/	Plasma FA and carnitine	Reduced levels of plasma carnitine and plasma DHA, AA, linolenic and linoleic acids were found in 66%, 62%, 60%, 43% and 38%, respectively of ASD children. 54% of ASD patients had elevated ω6/ω3 ratio. ASD patients with GI manifestations had significantly increased percentage of reduced serum carnitine (91.7%) and plasma DHA levels (87.5%) than HC (respectively, 42.3%; 38.5%), (respectively, *p* < 0.001; *p* < 0.001).
Yui et al., 2016 [61]	Cross-sectional study	28 ASD subjects: Mean age 13.5 ± 4.6 years 71.43% males 21 HC: Mean age 13.9 ± 5.7 years 71.43% males	/	Plasma FA	Plasma EPA, DHA and arachidic acid levels and plasma DHA/AA and EPA/AA ratios were significantly higher in ASD compared to HC (respectively, *p* = 0.02; *p* = 0.03; *p* = 0.04; *p* = 0.0002; *p* = 000). Plasma AA and adrenic acid were significantly lower in ASD compared to HC (respectively, *p* = 0.05; *p* = 0.04).
Yui et al., 2016 [62]	Cross-sectional study	30 ASD subjects: Mean age 13.6 ± 4.3 years 33.33% males 20 HC: Mean age, 13.2 ± 5.4 years 30% males	/	Plasma FA	The plasma levels of EPA and the plasma ratios of EPA/AA and DHA/AA were significantly higher (respectively, *p* = 0.1; *p* = 000; *p* = 0.000), while the plasma levels of AA and metabolites, such as adrenic acid, were significantly lower in ASD compared to HC (respectively, *p* = 0.01; *p* = 0.0; *p* = 0.004).
Parletta et al., 2016 [63]	Cross-sectional study	85 ASD children 401 ADHD children 79 HC	/	Plasma FA	Children with ADHD and ASD had lower DHA, EPA and AA, higher AA/EPA ratio and lower ω3/ω6 than controls (*p* < 0.001 except AA between ADHD and controls: *p* = 0.047). Children with ASD had lower DHA, EPA and AA than children with ADHD (*p* < 0.001). Childhood Autism Rating Scale scores correlated significantly with DHA, EPA and AA (respectively, *p* = 0.002; *p* = 0.038; *p* = 0.021).
Puig-Alcatraz et al., 2016 [64]	Cross-sectional study	26 ASD children aged 4–13 years 23 HC aged 4–12 years	/	Urinary adipic acid, suberic acid	No increase in the concentration of adipic acid or suberic in children with ASD compared to HC. The increase in adipic acid concentration was significantly and indirectly correlated with the severity of the deficit in socialization and communication skills in ASD children.
Wang et al., 2016 [65]	Cross-sectional study	73 ASD children: Mean age 4.6 ± 0.8 years 80.82% males 63 HC: Mean age 4.1 ± 0.7 years 80.95% males	/	Plasma metabolomics	ASD was associated with 2 metabolites: sphingosine 1-phosphate and DHA (respectively, *p* < 0.001; *p* < 0.001).
Yui et al., 2016 [66]	Cross-sectional study	30 ASD subjects: Mean age 13.0 years 20 sex-matched HC: Mean age 13.6 years	/	Plasma FA	ASD had significantly higher plasma DHA/AA and EPA/AA ratios compared to HC. The plasma ceruloplasmin levels in ASD were significantly reduced compared to HC. Multiple linear regression demonstrated that plasma DHA/AA ratio was a fitting model for distinguishing ASD from the HC.
Cariou et al., 2018 [67]	Cross-sectional study	839 adult psychiatric patients: group 1: hypobetalipoproteinemia (HBL) Mean age 35 ± 10 years 65% male group 2: non-HBL Mean age 44 ± 14 years 59% male	/	Plasma TC, HDL-C, LDL-C, TG	Psychiatric patients with HBL were characterized by a higher frequency of specific developmental disorders (including autism) (*p* = 0.011).
Howsmon et al., 2018 [68]	Cross-sectional study	63 ASD children: Median age 7.8 years: 49 HC: Median age 10.0 years	/	RBC membranes FA composition	FA do not allow for classification at the individual level.
Toscano et al., 2018 [69]	Clinical trial	64 ASD children aged 6–12 years: experimental group: *n* = 46 control group: *n* = 18	48-week exercise-based intervention	Plasma TC, HDL, LDL	The experimental group showed beneficial effects on metabolic indicators (TC, HDL, LDL), autism traits and parent-perceived quality of life.
Benachenhou et al., 2019 [70]	Cross-sectional study	79 ASD children: Mean age 19.4 ± 12.1 years 81% male 79 HC: Mean age 19.4 ± 12.0 years 81% male	/	Plasma TC, HDL-C, TG, LDL-C	TC levels below the 10th centile were associated with a higher rate of ASD-associated ID (OR = 3.33; 95% CI: 1.26–8.00) and anxiety/depression (OR = 4.74; 95% CI: 1.40–15.73).
Hassan et al., 2019 [71]	Cross-sectional study	63 ASD children 63 age- and sex-matched HC	/	Plasma TC	The serum levels of TC was significantly lower among ASD when compared with HC (*p* < 0.05).
Blazewicz et al., 2020 [33]	Clinical trial	57 ASD children: 100% male group 1: low-fat diet (LFD), *n* = 14 Mean age 16.6 years group 2: gluten-casein-free diet (GF-CF), *n* = 10 Mean age 17.8 years group 3: regular diet (RD), *n* = 35 Mean age 17.3 years 36 HC: Mean age 17.6 years 100% male RD	Different type of diet: LFD, GF-CF, RD	Plasma CRP, TC, HDL-C, TG	First assessment: in ASD subjects compared to HC: increased BMI, CRP and TC/HDL and decreased HDL-C for all types of diets (*p* < 0.05) increased TG in the group of LFD (*p* = 0.003) and RD individual (*p* < 0.001) increased non-HDL-C in the group of GF–CF (*p* = 0.008) and RD subjects (*p* < 0.001) Second assessment: increased levels of TC, non HDL-C and TC/HDL and decreased level of HDL-C for all ASD individuals regardless of diets used (*p* < 0.05) BMI and CRP increased only for individuals on LFD (BMI: *p* = 0.029; CRP: *p* < 0.001) and RD (BMI: *p* < 0.001; CRP: *p* > 0.001)
Usui et al., 2020 [72]	Cross-sectional study	152 ASD children 122 HC	/	Plasma FA, lipoprotein analysis	48 metabolites were identified in the plasma of ASD children by lipidomics (linoleic acid: *p* = 0.0133; linolenic acid: *p* = 0.0141; EPA: *p* = 0.0147; oleic acid: *p* = 0.0284; EPA: *p* = 0.0327; AA: *p* = 0.0395). Among these, increased FA, such as ω3 and ω6, showed correlations with clinical social interaction score and ASD diagnosis (*p* < 0.05). Specific reductions of plasma VLDL and APOB in ASD were found by large-scale lipoprotein analysis.
Yui et al., 2020 [73]	Cross-sectional study	11 ASD subjects: Mean age 12.3 ± 5.4 years 27.27% males 7 HC: Mean age 10.0 ± 4.1 years 57.14% males	/	Plasma FA, MDA-LDL, superoxide dismutase	Plasma levels of MDA-LDL, EPA, DHA and DHA/AA ratios were significantly higher, while plasma superoxide dismutase levels were significantly lower in ASD than in HC (respectively, *p* = 0.034; *p* = 0.000; *p* = 0.000; *p* = 0.000; *p* = 0.006). Multiple linear regression and adaptative Lasso analysis revealed association of increased plasma DHA levels with the Aberrant Behavior Checklists scores and increased plasma MDA-LDL levels.

**Table 2:** Associations between serum/plasma total cholesterol (TC) levels and Autism spectrum disorders.

TC Serum/Plasma Levels	Increased	Decreased
	Dziobek et al., 2005 (in Asperger syndrome)—cross-sectional study [42]	Goldenberg et al., 2003—cross-sectional study [40]
	Blazewicz et al., 2020—prospective study [33]	Tierney et al., 2006—cross-sectional study [45]
		Schengrund et al., 2012—cross-sectional study [52]
		Hassan et al., 2018—cross-sectional study [71]
		Benachenhou et al., 2019—cross-sectional study [70]

**Table 3:** Associations between serum/plasma ω6/ω3 ratio and Autism spectrum disorders.

ω6/ω3 Serum/Plasma Ratio	Increased	Decreased
	Vancassel et al., 2001—cross-sectional study [39]	Jory, 2015—cross-sectional study [59]
	Ghezzo et al., 2013—cross-sectional study [54]	Sliwinski et al., 2006—cross-sectional study [44]
	Esparham et al., 2015—cross-sectional study [58]	
	Mostafa and Al-Ayadhi, 2015—cross-sectional study [60]	

**Table 4:** Associations between docosahexaenoic acid (DHA) serum/plasma levels and Autism spectrum disorders.

DHA Serum/Plasma Levels	Increased	Decreased
	Bell et al., 2004—prospective trial [41]	Meguid et al., 2008—clinical trial [46]
	Sliwinski et al., 2006—cross-sectional study [44]	Wiest et al., 2009—cross-sectional study [48]
	Esparham et al., 2015—cross-sectional study [58]	Ghezzo et al., 2013—cross-sectional study [54]
	Yui et al., 2016—cross-sectional study [61]	Brigandi et al., 2015—cross-sectional study [57]
	Yui et al., 2016—cross-sectional study [62]	Jory, 2015—cross-sectional study [59]
	Yui et al., 2020—cross-sectional study [73]	Mostafa and Al-Ayadhi, 2015—cross-sectional study [60]
		Parletta et al., 2016—cross-sectional study [63]
		Wang et al., 2016—cross-sectional study [65]

**Table 5:** Associations between arachidonic acid (AA) serum/plasma levels and Autism spectrum disorders.

AA Serum/Plasma Levels	Increased	Decreased
	Yui et al., 2016—cross-sectional study [62]	Bell et al., 2004 – prospective trial [41]
	Yui et al., 2016—cross-sectional study [66]	Meguid et al., 2008—clinical trial [46]
		Brigandi et al., 2015—cross-sectional study [57]
		Jory, 2015—cross-sectional study [59]
		Mostafa and Al-Ayadhi, 2015—cross-sectional study [60]
		Yui et al., 2016—cross-sectional study [61]
		Parletta et al., 2016—cross-sectional study [63]

**Table 6:** Associations between eicosapentaenoic acid (EPA) serum/plasma levels and Autism spectrum disorders.

EPA Serum/Plasma Levels	Increased	Decreased
	Bell et al., 2004—prospective trial [41]	Ghezzo et al., 2013—cross-sectional study [54]
	Yui et al., 2016—cross-sectional study [61]	Parletta et al., 2016—cross-sectional study [63]
	Yui et al., 2016—cross-sectional study [62]	

## Data Availability

Not applicable.

## Abbreviations

AA	arachidonic acid
APOB	apoprotein B
ASD	autism spectrum disorder
BMI	body mass index
CE	cholesterol ester
CRP	C-reactive protein
CS	cholesterol sulfate
DD	developmental delay
DHA	docosahexaenoic acid
EPA	eicosapentaenoic acid
FA	fatty acids
FAD	fatty acids deficiency
FS	free sterol
GF-CF	gluten-casein-free diet
GI	gastrointestinal
GM1	ganglioside GM1
HBL	hypobetalipoproteinemia
HC	healthy controls
HDL-C	high-density lipoprotein
ID	intellectual disabilities
LDL-C	low-density lipoprotein
LFD	low-fat diet
MDA-LDL	malondialdehyde-modified low-density lipoprotein
PC	phosphatidylcholine
PE	phosphatidylethanolamine
PS	phosphatidylserine
PUFA	polyunsaturated fatty acids
RBC	red blood cell
RD	regular diet
SLOS	Smith-Lemli-Opitz Syndrome
TC	total cholesterol
TG	triglycerides
VLDL	very-low-density lipoprotein
7-DHC	7-dehydrocholesterol
8-DHC	8-dehydrocholesterol
